# Platelet Reactivity Is Independent of Left Atrial Wall Deformation in Patients with Atrial Fibrillation

**DOI:** 10.1155/2016/9754808

**Published:** 2016-03-16

**Authors:** Nathan Procter, Vincent Goh, Gnanadevan Mahadevan, Simon Stewart, John Horowitz

**Affiliations:** ^1^Basil Hetzel Institute, The Queen Elizabeth Hospital, The University of Adelaide, Woodville South, SA 5011, Australia; ^2^National Health and Medical Research Council Centre of Research Excellence to Reduce Inequality in Heart Disease, Mary MacKillop Institute for Health Research, Australian Catholic University, Melbourne, VIC 3065, Australia

## Abstract

It has been documented recently that left atrial (LA) deformation in AF patients (while in AF) is predictive of subsequent stroke risk. Additionally, diminished LA deformation during AF correlates with the presence of LA blood stasis. Given that endothelial function is dependent on laminar blood flow, the present study sought to investigate the effect of diminished LA deformation (during AF) on platelet reactivity and inflammation in AF patients. Patients (*n* = 17) hospitalised with AF underwent echocardiography (while in AF) for determination of peak positive LA strain (LASp). Whole blood impedance aggregometry was used to measure extent of ADP-induced aggregation and subsequent inhibitory response to the nitric oxide (NO) donor, sodium nitroprusside. Platelet thioredoxin-interacting protein (Txnip) content was determined by immunohistochemistry. LASp tended (*p* = 0.078) to vary inversely with CHA_2_DS_2_VASc scores. However, mediators of inflammation (C-reactive protein, Txnip) did not correlate significantly with LASp nor did extent of ADP-induced platelet aggregation or platelet NO response. These results suggest that the thrombogenic risk associated with LA stasis is independent of secondary effects on platelet aggregability or inflammation.

## 1. Introduction

Atrial fibrillation (AF) is increasing in global prevalence and carries with it incremental thromboembolic risk. Currently, this risk is assessed through the application of clinical algorithms such as the CHA_2_DS_2_VASc score [1], from which appropriate antithrombotic therapy may be determined.

The precise pathophysiology underpinning the utility of the CHA_2_DS_2_VASc score in predicting thromboembolic events continues to be an ongoing area of investigation: one such facet of this interest is the association of inflammation with thromboembolic risk. Just as inflammation is a pivotal mediator of the pathogenesis of AF [2], it also appears to be involved in thromboembolic risk. Plasma concentrations of the inflammatory mediator Galectin-3 correlated positively [3] and those of the anti-inflammatory mediator adiponectin correlated inversely [4], with CHA_2_DS_2_VASc scores. The potential utility of these biomarkers in refining the prediction of thromboembolic risk continues to be explored.

An alternative approach to predicting thromboembolic risk relies ultimately on the concept that avoiding stasis of regional wall activity is protective [5]: thus the focus is to investigate the potential of extent of atrial wall motion for predictive power for incidence of thromboembolism. Recently, two-dimensional speckle tracking echocardiography was used to evaluate the extent of left atrial deformation (peak positive left atrial strain, LASp) experienced by patients in AF, correlating diminished LASp with incidence of stroke [6]. Decreased LASp has also been associated with extent of left atrial fibrosis [7], incidence of new onset AF [8], and the occurrence of left atrial blood stasis and thrombus formation [9].

However, it is also possible that the maintenance of left atrial deformation limits rheological stimuli towards thrombosis: nonlaminar blood flow has variously been associated with uncoupling of endothelial nitric oxide (NO) synthase [10], generation of reactive oxygen species [11], and increased expression of the proinflammatory mediator thioredoxin-interacting protein (Txnip) [12, 13]. Given the aforementioned propensity for blood stasis in the presence of diminished atrial wall motility and associated risk for thromboembolism, the present study sought to investigate potential intersections of LASp and platelet reactivity, as well as the involvement of the inflammatory mediators C-reactive protein (CRP) and Txnip, in a cohort of AF patients.

## 2. Methods

### 2.1. Study Population

Patients (*n* = 17) were included as a prospectively defined subset of the Standard versus Atrial Fibrillation Specific Management Study (SAFETY) [14, 15]. Inclusion and exclusion criteria for SAFETY have been reported previously [14]. Patients receiving P2Y_12_ receptor antagonist therapy were also excluded due to the impact such agents have on platelet ADP response. All patients underwent transthoracic echocardiography while in AF.

### 2.2. Clinical

All patients underwent routine clinical and biochemical investigation upon hospital admission. Standard echocardiography including Doppler studies was performed according to established guidelines [16]. Left ventricular ejection fraction was determined using Simpson's biplane method. Measurement of left atrial wall motion was performed as reported by Shih et al. [6], intraobserver coefficient of variation (CV) was 8.3%, and interobserver CV was 5.1%. Mean heart rate during transthoracic echocardiography was 84 ± 12 bpm.

### 2.3. Laboratory

*Platelet aggregometry* was performed using whole blood impedance aggregometry as previously described [17]. Briefly, venous blood was collected from an antecubital vein into 10 mL tubes containing 1 : 10 volume of acid citrate anticoagulant (2 parts of 0.1 M citric acid to 3 parts of 0.1 M trisodium citrate). Aggregation was induced with ADP (2.5 *μ*M), and responses were recorded for electrical impedance (Ω) via a computer interface system (Aggrolink, Chrono-Log, Havertown, Pennsylvania, USA). The NO donor sodium nitroprusside (SNP, 10 *μ*M) was used to measure platelet response to NO. Inhibition of aggregation by SNP was evaluated as percentage of maximal aggregation in the absence of SNP. In order to minimize inaccuracies in calculation of inhibitory effect of SNP, at least 4 Ω of ADP response was required.

### 2.4. Statistical Methods

All data for normally distributed parameters are expressed as mean ± standard deviation unless otherwise stated. Skewed data are expressed as median and interquartile range (IQR). Where applicable, nonnormal distributions were transformed using Log or Ln functions. Univariate correlates of LASp were sought by linear regression. Data were analyzed using the IBM SPSS Statistics 20 and GraphPad Prism 6 software packages.

## 3. Results

The clinical and pharmacological profiles of the study cohort can be observed in Tables 1 and 2, respectively. A total of 8 patients were excluded from analysis because of inadequate quality of LASp determination from echocardiographic records. Patients were typical for an AF population, being elderly, of moderate stroke risk on the basis of clinical scoring algorithms, and with relatively preserved renal function. The majority of patients were receiving oral anticoagulant (warfarin) therapy and renin-angiotensin-aldosterone system blockade (angiotensin receptor antagonists or angiotensin-converting enzyme antagonists), while approximately 1/3 were on lipid-lowering therapies (statins). Echocardiographic parameters of interest are summarised in Table 3.

**Figure 1:**
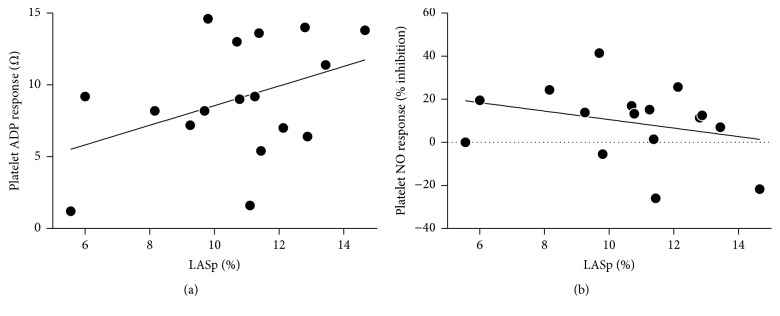
Relationships between peak positive left atrial strain (LASp) and (a) ADP-induced platelet aggregation (*r* = 0.408, *p* = 0.104) or (b) platelet response to NO (*r* = −0.290, *p* = 0.275).

**Figure 2:**
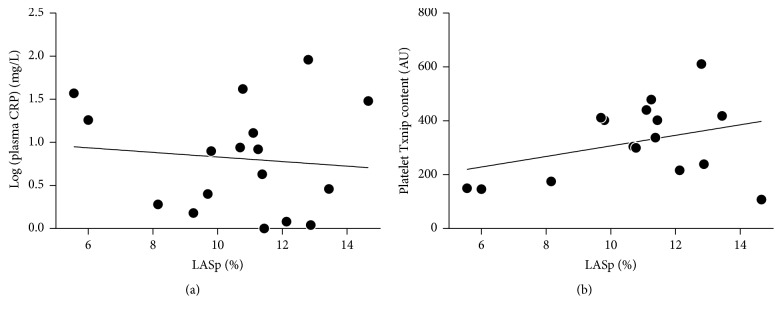
No significant correlation was observed between LASp and (a) plasma CRP concentrations (*r* = −0.105, *p* = 0.689) or (b) platelet Txnip content (*r* = 0.345, *p* = 0.191).

**Figure 3:**
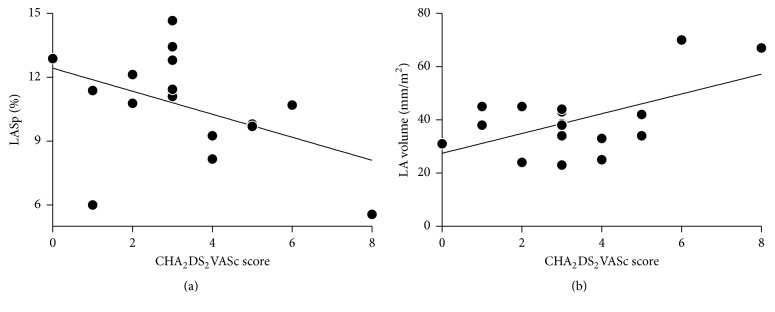
(a), An inverse trend between LASp and CHA_2_DS_2_VASc score was nonsignificant (*r* = −0.44, *p* = 0.08). (b) CHA_2_DS_2_VASc score and indexed LA volume were directly correlated (*r* = 0.56, *p* < 0.05).

The extent of atrial wall motility during AF bore no significant relationship with platelet reactivity (Figure 1): correlations between LASp and ADP-induced platelet aggregation, or LASp and platelet response to NO, were both nonsignificant. It should be noted, indeed, that if anything, there was an association (*p* = 0.104) between increasing LASp and increasing ADP-induced platelet aggregation. Similarly, LASp was not significantly associated with plasma CRP concentrations nor with platelet Txnip content (Figure 2). A trend towards an inverse association between CHA2DS2VASc scores and LASp was observed, while CHA2DS2VASc scores and indexed LA volume were directly correlated (Figure 3).

## 4. Discussion

The current study, unique in the literature to date, has suggested that LASp may vary inversely with clinical indices of stroke risk, such as CHA_2_DS_2_VASc score. However, it has failed to demonstrate any evidence that maintained LASp might protect against thromboembolism by restoring homeostasis as regards the inflammatory and autacoid bases for thrombosis.

Patients with AF are particularly susceptible to the development of thrombi in their left atrium, with the presence of left atrial blood stasis, a significant factor in their formation [18, 19]. Similarly, left atrial stasis has been correlated with clinical risk factors and stroke incidence [5, 20]. Such thrombi are commonly platelet-rich in their morphology [21]; while not correlated with clinical measures of thromboembolic risk [22, 23], the platelet hyperaggregability that is present in patients with AF [24, 25] nonetheless would seemingly have an integral role in the formation of such thrombi.

One of the factors contributing to platelet hyperaggregability in this context is potential impairment of the NO signaling pathway (see [26] for review). Effective generation of NO by endothelial NO synthase is impaired by the presence of nonlaminar blood flow, such as what occurs in the presence of AF [11, 27, 28]. In theory, the extent of atrial motility present during AF may help preserve the functioning of this pathway through amelioration of blood stasis and, as such, would be reflected through measures of platelet reactivity. However, in our investigation we observed no such correlation between atrial wall movement and platelet response to ADP or NO. Similarly, inflammation is regulated in part by NO [29, 30]; specifically Txnip expression is suppressed by the presence of NO [31, 32]. Yet no correlation was observed between atrial wall motion and markers of inflammation in the current investigation.

One potential cause for error in acquisition of LASp data is variable degrees of tachycardia in the LA [6]. This problem was minimized because the actual heart rate during LASp measurement was 84 ± 12 beats per minute (bpm) and because multiple views of the LA were incorporated. Reproducibility of LA strain estimates in AF patients is best achieved by considering only periods of relative heart rate stability [33]. No particular precautions were taken in this regard, and this therefore constitutes a potential limitation on accuracy of data.

The major limitation of the current study is the potential for type 2 error. However, it seems unlikely from the current data that any strong correlation has been missed, as regards the measured physiological parameters. However, it is possible that platelet responsiveness to NO may not reflect that of the endocardial endothelium.

According to the principles outlined by Virchow's triad [34], the presence of blood stasis in the atria during AF is a predisposing factor for thrombogenesis. The current data dissociate this stasis from increased platelet reactivity and/or inflammation. Given that biomechanical (such as LASp), as opposed to biochemical, factors are the primary determinants of left atrial thrombogenesis, it would be expected that effective maintenance of sinus rhythm in AF patients would significantly reduce their thromboembolic risk through improved atrial flow.

## Figures and Tables

**Table 1:** Clinical profile of the study cohort.

Sociodemographic profile (*n* = 17)	
Gender, *n* (% male)	9 (52.9)
Age (yrs)	72 ± 12
Aged ≥ 75 years, *n* (%)	9 (52.9)
Comorbidities	
Congestive heart failure, *n* (%)	2 (11.8)
Hypertension, *n* (%)	11 (64.7)
Diabetes mellitus, *n* (%)	4 (23.5)
Prior stroke/TIA, *n* (%)	4 (23.5)
Clinical presentation	
Admission heart rate (bpm)	98 ± 31
Plasma creatinine (*μ*M)	86 ± 22
Plasma CRP (mg/L)	16.0 ± 23.3
CHA_2_DS_2_VASc score	3.1 ± 2.0

**Table 2:** Pharmacological therapy present in the study cohort.

Pharmacological profile (*n* = 17)	
Antithrombotic therapy	
Aspirin, *n* (%)	4 (23.5)
Warfarin, *n* (%)	13 (76.5)
Rate and/or rhythm control therapy	
Antiarrhythmics, *n* (%)	5 (29.4)
*β* receptor antagonist, *n* (%)	11 (64.7)
RAAS inhibitors	
ACE inhibitor, *n* (%)	9 (52.9)
Angiotensin receptor antagonist, *n* (%)	5 (29.4)
Other medications	
Statin, *n* (%)	6 (35.3)

**Table 3:** Echocardiographic profile of the study cohort.

Echocardiographic profile (*n* = 17)	
Indexed left atrial volume (mm·m^−2^)	39.7 ± 13.0
Left atrial emptying fraction (%)	21.9 ± 11.0
Peak positive left atrial strain (%)	10.6 ± 2.4
Left ventricular emptying fraction (%)	53.7 ± 7.3
